# Standards for the Use of Enteral Nutrition in Patients with Diabetes or Stress Hyperglycaemia: Expert Consensus

**DOI:** 10.3390/nu15234976

**Published:** 2023-11-30

**Authors:** María I. Rebollo-Pérez, Luna Florencio Ojeda, Pedro P. García-Luna, José A. Irles Rocamora, Gabriel Olveira, Juan Ramón Lacalle Remigio, Carmen Arraiza Irigoyen, Alfonso Calañas Continente, Cristina Campos Martín, María Luisa Fernández Soto, José Manuel García Almeida, María Laínez López, Concepción Losada Morell, Luis Miguel Luengo Pérez, Teodosia Muñoz de Escalona Martínez, José L. Pereira-Cunill, Francisco J. Vílchez-López, Juana M. Rabat-Restrepo

**Affiliations:** 1 Endocrinology and Nutrition Clinical Management Unit, University Hospital Juan Ramón Jiménez, 21005 Huelva, Spain; misabel.rebollo.sspa@juntadeandalucia.es (M.I.R.-P.); lunaflorencio90@gmail.com (L.F.O.); marialainezlopez@yahoo.es (M.L.L.); 2 Regional Andalusian Health Service, Service of Endocrinology and Nutrition, University Hospitals Virgen del Rocío, 41013 Seville, Spain; garcialunapp@yahoo.es (P.P.G.-L.); jpereira@cica.es (J.L.P.-C.); 3 Faculty of Medicine, University of Seville, 41009 Seville, Spain; irles@us.es (J.A.I.R.); rabat@us.es (J.M.R.-R.); 4 Endocrinology and Nutrition Clinical Management Unit, University Hospital Valme, 41014 Seville, Spain; 5 Biomedical Research Institute of Málaga (IBIMA), 29010 Málaga, Spain; jgarciaalmeida@gmail.com; 6 Endocrinology and Nutrition Clinical Management Unit, Regional University Hospital of Málaga/University of Málaga, 29010 Málaga, Spain; 7 Biomedical Network Research Centre for Diabetes and Associated Metabolic Diseases (CIBERDEM) (CB07/08/0019), Health institute Carlos III, 28029 Madrid, Spain; 8 Medicine and Dermatology Department, Faculty of Medicine, University of Málaga, 29010 Málaga, Spain; 9 University Hospital of Jaén, 23007 Jaén, Spain; carmenarraizairigoyen@gmail.com; 10 Endocrinology and Nutrition Clinical Management Unit, University Hospital Reina Sofia, 14004 Córdoba, Spain; contentine@gmail.com; 11 Endocrinology and Nutrition Clinical Management Unit, University Hospital Virgen Macarena, 41009 Seville, Spain; 12 Endocrinology and Nutrition Clinical Management Unit, University Hospital San Cecilio, 18012 Granada, Spain; mlfernan@ugr.es; 13 Biosanitary Institute of Granada, Medicine Department, Faculty of Medicine of Granada, University of Granada, 18010 Granada, Spain; 14 Endocrinology and Nutrition Clinical Management Unit, University Hospital Virgen de la Victoria, 29010 Málaga, Spain; 15 Endocrinology and Nutrition Clinical Management Unit, Internal Medicine Clinical Management Unit, Hospital Infanta Margarita, 14940 Cabra, Córdoba, Spain; conchalos19@telefonica.net; 16 University Hospital of Badajoz, 06080 Badajoz, Spain; luismluengo@unex.es; 17 Endocrinology and Nutrition Clinical Management Unit, University Hospital Torrecárdenas, 04009 Almeria, Spain; tmescalonam@gmail.com; 18 Endocrinology and Nutrition Clinical Management Unit, Biomedical Research and Innovation Institute of Cádiz, University Hospital Puerta del Mar, 11009 Cádiz, Spain; franvilchez1977@gmail.com

**Keywords:** enteral nutrition, diabetes, stress hyperglycaemia, expert consensus, Delphi

## Abstract

(1) Background: Hyperglycaemia that occurs during enteral nutrition (EN) should be prevented and treated appropriately since it can have important consequences for morbidity and mortality. However, there are few quality studies in the literature regarding the management of EN in this situation. The objective of this project was to attempt to respond, through a panel of experts, to those clinical problems regarding EN in patients with diabetes or stress hyperglycaemia (hereinafter referred to only as hyperglycaemia) for which we do not have conclusive scientific evidence; (2) Methods: The RAND/UCLA Appropriateness Method, a modified Delphi panel method, was applied. A panel of experts made up of 10 clinical nutrition specialists was formed, and they scored on the appropriateness of EN in hyperglycaemia, doing so in two rounds. A total of 2992 clinical scenarios were examined, which were stratified into five chapters: type of formula used, method of administration, infusion site, treatment of diabetes, and gastrointestinal complications. (3) Results: consensus was detected in 36.4% of the clinical scenarios presented, of which 23.7% were deemed appropriate scenarios, while 12.7% were deemed inappropriate. The remaining 63.6% of the scenarios were classified as uncertain; (4) Conclusions: The recommendations extracted will be useful for improving the clinical management of these patients. However, there are still many uncertain scenarios reflecting that the criteria for the management of EN in hyperglycaemia are not completely standardised. More studies are required to provide quality recommendations in this area.

## 1. Introduction

Medical nutrition therapy (enteral and parenteral) is one of the most recognised causes of hyperglycaemia in the hospital setting, along with other factors such as stress due to illness or the use of hyperglycaemic drugs. The degree of hyperglycaemia is related to the severity of the disease and is an important prognostic marker [1,2].

Expert recommendations recognise that there are few studies that evaluate the prevalence of hyperglycaemia in patients receiving EN, with reported values ranging from 30% to 47% and with half of the patients lacking a previous diagnosis of diabetes mellitus (DM) [3,4]. Abuin-Fernandez (2020) estimated the prevalence of DM to be 31.8% for patients receiving home enteral nutrition (HEN) through a feeding tube [5]. For patients receiving medical nutrition therapy, hyperglycaemia has been associated with increased morbidity and mortality [6,7].

At the time this project began, the evidence from clinical trials on the management of hyperglycaemia in patients receiving EN was scant, coming predominantly from studies conducted on specific EN formulas [8,9].

Given the lack of evidence, professionals must resort to other sources to guide their clinical practice, including consensus statements that collect and systematise expert opinions. 

The objective of this project was to identify and respond to those problems for which we do not have scientific evidence in the use of EN for patients with hyperglycaemia, through an expert consensus, using the RAND/UCLA Appropriateness Method. 

## 2. Materials and Methods

The consensus method—the RAND/UCLA Appropriateness Method (RAM) [10]—was used to establish criteria for the appropriate use of medical treatments. 

Decisions about the design of the project were the responsibility of the steering group, which consisted of professionals with experience in clinical nutrition (M.I.-R., J.M.-R., P.P.-G.L., J.A.-I., and G.-O.), with methodological support (J.R.-L.R.). 

The phases and tasks carried out to develop the panel were completed in accordance with what is indicated in the RAM [11] manual, and they are summarised below:

### 2.1. Literature Review

A bibliographic search of articles on the management of EN in hyperglycaemia was carried out. The search was performed in PubMed, filtering for clinical trials, meta-analyses, systematic reviews, and expert recommendations, with the keywords enteral nutrition AND diabetes or enteral nutrition AND hyperglycaemia. The steering group selected publications that, in their opinion, could serve as support for the panel discussions.

### 2.2. Preparation of the Clinical Scenarios or Indicatios

A questionnaire was prepared (Appendix A), and it comprised 32 open questions about nutrient intake targets, administration regimens, the use of specific EN formulas for hyperglycaemia or other concomitant specific pathologies, the treatment of hyperglycaemia in patients with EN and the management of complications of EN. The questionnaire was sent by email to 11 specialists in clinical nutrition from hospitals in Andalusia and Extremadura (panel members). The responses were used to identify the most relevant clinical variables for decision-making on the use of EN in hyperglycaemia (collected in Appendix B).

**Figure 1:**
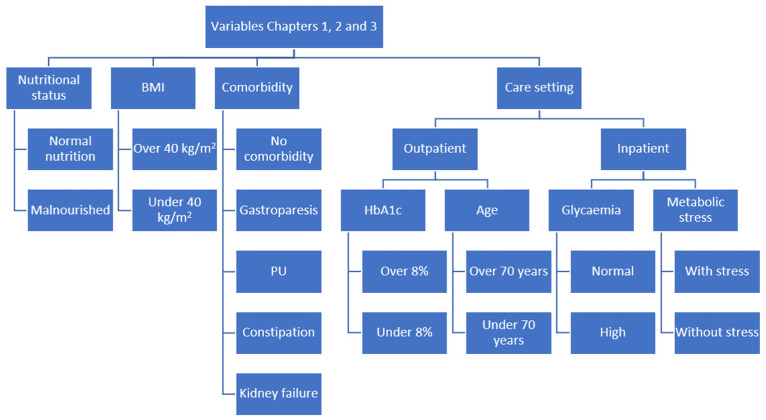
Organisation chart of the variables used to build the clinical scenarios for Chapters 1, 2 and 3. BMI: body mass index; PU: pressure ulcer; HbA1c: glycosylated haemoglobin.

**Figure 2:**
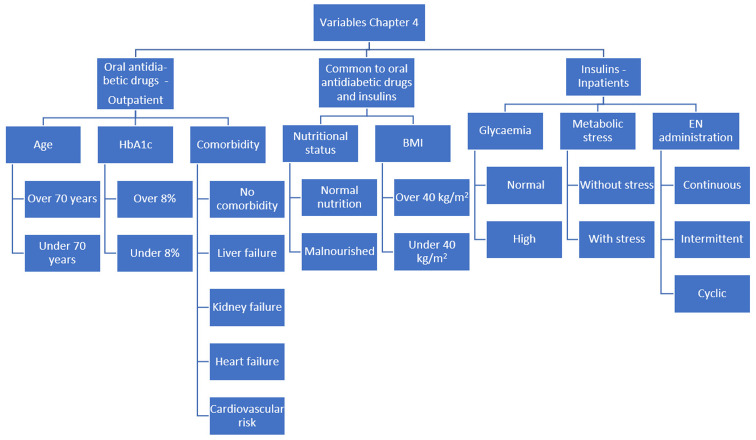
Flowchart of the variables used to build the clinical scenarios for Chapter 4. BMI: body mass index; HbA1c: glycosylated haemoglobin; EN: enteral nutrition.

**Figure 3:**
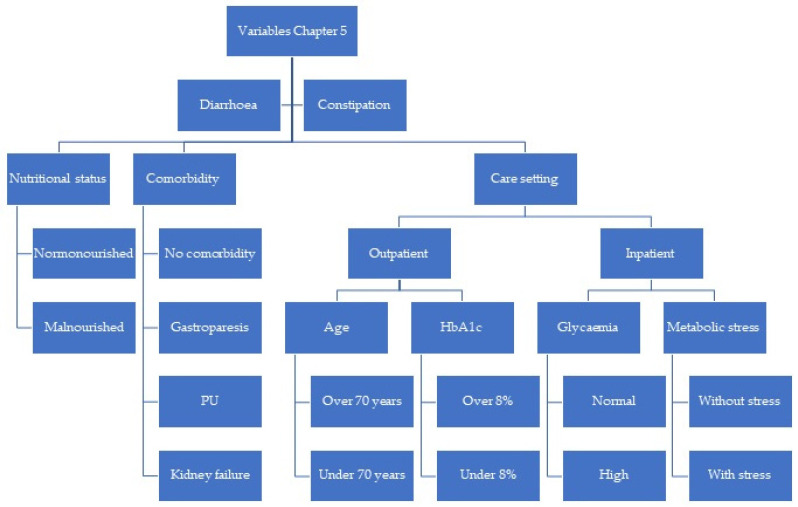
Flowchart of the variables used to build the clinical scenarios for Chapter 5. BMI: body mass index; PU: pressure ulcer; HbA1c: glycosylated haemoglobin; EN: enteral nutrition.

**Figure 4:**
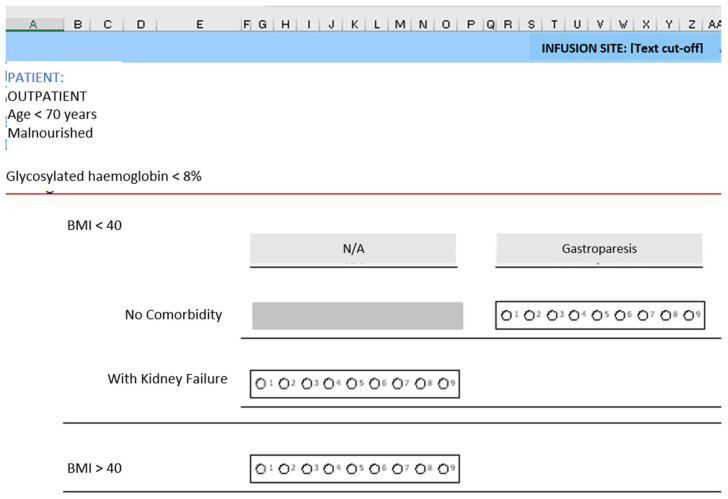
Fragment of one of the spreadsheets with the scenarios and scales used to rate appropriateness.

The clinical scenarios were created by combining the categories of those variables (Figure 1, Figure 2 and Figure 3). The steering group reviewed all the scenarios and eliminated those that they considered not to occur in clinical practice. Finally, a total of 2992 clinical scenarios were included. Figure 4 shows the appearance of some of the scenarios, as they were presented to the panel members. 

The scenarios were grouped into chapters according to the clinical variables chosen to assess a specific aspect of EN use. For this reason, the number of scenarios is different in each chapter.

The contents of the chapters are listed below:

- Chapter 1: types of formulas used (768 clinical scenarios);
- Chapter 2: method of administration (288 clinical scenarios);
- Chapter 3: infusion site (192 clinical scenarios);
- Chapter 4: treatment of diabetes (1104 clinical scenarios);
- Chapter 5: management of complications (640 clinical scenarios).

### 2.3. Selection of the Members of the Panel of Experts

The 10 members of the panel were selected from the nutrition specialists who were working in public hospitals in Andalusia and Extremadura, and all of them had more than 10 years of experience and spent at least 80% of their working time in clinical nutrition.

### 2.4. Rounds to Score Indications

The panellists scored each scenario anonymously in two rounds and at different times. In the second round, the panellists were provided with information about the responses of the other panellists in the first round, along with the panellist’s own score. This information is explained below.

#### 2.4.1. Definition of Appropriateness

The panellists expressed their opinions in each scenario by scoring on a nine-point Likert-type scale that ranged from one (very inappropriate) to nine (very appropriate). The scores reflected how appropriate, according to the panellist, the use of the procedure was in that scenario. A procedure was considered appropriate if, in using the procedure, the potential benefits clearly outweighed the negative consequences that could occur. Conversely, the use of a procedure was inappropriate if the expected risks clearly outweighed the expected potential benefits. The benefits and risks were related to people’s health, regardless of financial or organisational costs.

#### 2.4.2. First Round

All panellists received the same documentation by email at the same time, and it included: the references selected after reviewing the literature on EN and hyperglycaemia; the definitions of the terms used in the list of indications (Appendix C); instructions for the scoring process; and the spreadsheet files containing the scenarios. In addition, they were given a list of the definitions of the variables used to create the scenarios.

#### 2.4.3. Second Round

**Figure 5:**
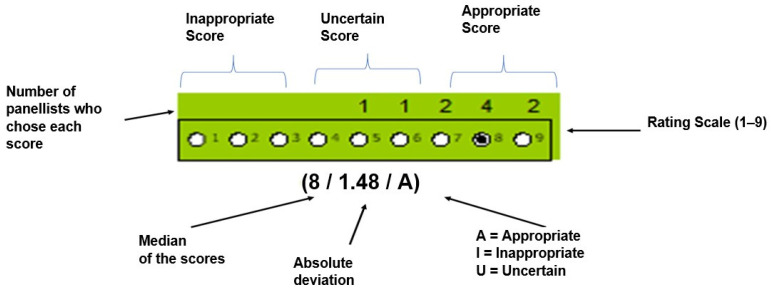
The appearance of a scenario with the results that were presented to a panellist indicating the elements that were represented.

Once all the scores from the first round were received and processed by us, a new document containing the scenarios was prepared, together with the results of the first round. This document was personalised for each panellist because the scores they had assigned in the first round were included. The key to interpreting the outputs and information that were presented to each panellist are shown in Figure 5. Colour coding was used to reflect whether an indication was rated as inappropriate (yellow), uncertain (pink) or appropriate (green).

Then, the panellists were invited to a face-to-face meeting in which each panellist received the aforementioned documents. During the session, the panellists discussed the results of the first round, anonymously scoring the same scenarios again. Two moderators led the discussions; their roles were limited to controlling the debates and they did not vote or influence the opinions of the panel members. 

As a result of the discussions, the panellists proposed that the scenarios for Chapters 4 and 5 should be modified. These modified scenarios were voted on in two new rounds, both of which were limited to those chapters. 

### 2.5. Statistical Criteria for Consensus

After each round, the scores of the panellists were statistically processed. As a result, in each scenario, the degree of agreement between the panellists and their opinions on the use of the procedure was measured.

It was defined that there was agreement within the panel if the scores of all the panellists, after excluding the highest and the lowest, were within the same three-point interval. It was defined that there was disagreement if four or more panellists voted in the interval from one to three and another four or more voted in the interval from seven to nine. Those scenarios that were not classified as agreement or disagreement were considered uncertain.

Finally, each indication was classified as one of the following categories according to the degree of agreement and the median of the scores:

- Appropriate indication if the median was in the interval from seven to nine, without disagreement.
- Uncertain indication if the median was in the interval from four to six, or when there was disagreement among the panellists, independent of the median;
- Inappropriate indication if the median was in the interval of one to three, without disagreement.

## 3. Results

Table 1, Table 2, Table 3, Table 4 and Table 5 summarise the main results of the panel, specifically those clinical scenarios in which there was consensus on both an appropriate and inappropriate indication, with some contributions resulting from the discussion of the panel members. 

The detailed results are reported in Appendix D. The full information on the scores for all scenarios is available upon request.

In Chapters 1–3, the scores of nine panellists were collected (the files of one panellist were unrecoverable). These chapters were discussed in a different session than the one held for Chapters 4 and 5, where there were 10 panellists. 

In Appendix E, the means of the median, the median absolute deviation, the proportion of agreement and disagreement, and the percentages of the scenarios rated as appropriate, inappropriate or uncertain are presented. 

In most of the scenarios, the panellists agreed (90.4%), although we found that agreement was reached because, above all, they voted in the interval from four to six, and those scenarios were classified as uncertain (63.6%). The rest of the scenarios (36.4%) were classified as appropriate (23.7%) or inappropriate (12.7%). 

**Figure 6:**
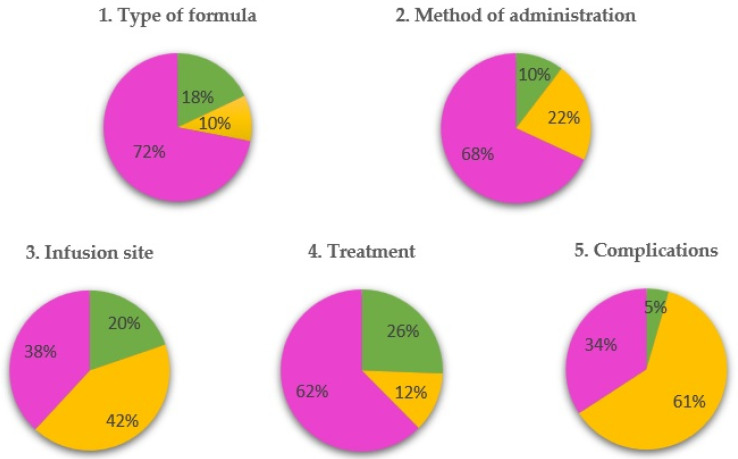
Proportion of appropriate, inappropriate and uncertain indications by chapter. Yellow represents inappropriate, pink represents uncertain, and green represents appropriate.

Figure 6 shows the proportions of the appropriate, inappropriate and uncertain scenarios for each chapter.

## 4. Discussion

The opinions expressed by experts in clinical nutrition in this work reflect that the criteria for the management of EN in hyperglycaemia are not standardised. Of the total presented scenarios, 36.4% were agreed upon as being appropriate or inappropriate. The most relevant results from each chapter are discussed below. 

### 4.1. Types of Formulas Used

Any EN formula can be used in diabetes or hyperglycaemia if the hypoglycaemic therapy is appropriately adjusted. However, most publications recommend the use of specific diets for diabetes since they seem to provide added value for the metabolic control of these patients compared to standard formulas [8,9,12,13].

Regarding the opinions of the panel experts on the use of different types of EN formulas in different clinical scenarios, the presence of pressure ulcers (PU) determined the appropriate indication of a specific high-protein formula for diabetes or of a specific formula for PU (formulas enriched with defined nutrients that promote healing, such as arginine), both for outpatients and inpatients. 

Coinciding with what was published [14], there was agreement that the use of a specific high-protein formula for diabetes was inappropriate in most scenarios with pre-dialysis kidney failure, with scenarios with associated metabolic stress arising as uncertain. This may have been due to the fact that patients with metabolic stress may present the protein-energy wasting syndrome associated with kidney failure and require the use of a high-protein formula in this situation. According to the latest recommendations of the American Diabetes Association (ADA), the recommended protein intake for patients with chronic kidney disease who are not on dialysis should be approximately 0.8 gr/kg weight/day [15]. In all the clinical scenarios with kidney failure, there was agreement in considering the specific formulas for this comorbidity to be appropriate. Some of these kidney-failure-specific enteral formulas include low-glycaemic-index carbohydrates, prebiotics and a high percentage of fat, and these may be appropriate for people with hyperglycaemia. 

There was also agreement that the specific high-calorie and high-protein formulas for diabetes would be inappropriate for inpatients with good glycaemic control and without metabolic stress, who also have a BMI of over 40 kg/m^2^, and for outpatients with normal nutrition, who also have a BMI of over 40 kg/m^2^. This may have been due to the calorie content of this type of formula. However, there was no agreement among the panellists regarding its use for patients with BMIs of over 40 kg/m^2^ who were inpatients with metabolic stress or who were malnourished outpatients. Although there are no good-quality clinical trials on this topic, current evidence suggests that low-calorie nutrition could improve outcomes for critically obese patients due to a lower rate of infectious complications and better control of hyperglycaemia. Thus, low-calorie and high-protein nutrition should be standard practices in the nutritional support of critically obese patients if there are no contraindications for it [16]. 

The panel experts agreed on the use of a specific normal-calorie and normal-protein formula for diabetes for patients with normal nutrition without additional comorbidities and who require EN on an outpatient basis. For outpatients, diabetes-specific formulas also improve metabolic control and, in some cases, HbA1c and insulin requirements compared to the use of a standard formula [4,13,17,18].

There was consensus on the inappropriate use of a standard formula for patients with poor glycaemic control, both for outpatients and inpatients, since the use of specific formulas for diabetes would be recommended in this group of patients, as mentioned above. The use of a standard formula with fibre would also be inappropriate in the case of gastroparesis, and the use of a standard formula without fibre would be inappropriate in the case of constipation. There was also agreement among the panel experts on the proper use of protein modules in the presence of PU and in malnourished outpatients with BMIs of over 40 kg/m^2^, although there is little scientific evidence on the use of protein modules in this clinical situation.

### 4.2. Method of Administration 

In the clinical practice guidelines for the management of EN in patients with hyperglycaemia, no specific recommendation is included for the most appropriate method of administration for these patients. The method was chosen, as for the non-diabetic population, by taking into account the underlying disease of the patient and their clinical situation. There was consensus among the experts on the appropriate use of continuous administration for hyperglycaemic inpatients with high glycaemia and metabolic stress. Thus, continuous infusions are generally used for patients who are critically ill and severely malnourished and for those who have been fasting for a long period of time or have been receiving parenteral nutrition [19]. The continuous administration of EN has been shown to decrease glucose levels in ventilated patients, attenuating glycaemic variability and decreasing insulin requirements, thus achieving better glycaemic control compared to intermittent administration [20].

In addition, there was consensus on the use of the continuous administration of EN for patients with gastroparesis; thus, in this subgroup of patients, the use of iso-osmolar formulas without fibre, with a slow initial EN infusion rate, is recommended, followed by progressively increasing the infusion rate until the calculated patient requirements are reached [21].

For a majority of the outpatients and stable inpatients (without metabolic stress), the administration of intermittent or bolus EN has been shown by experts to be appropriate, as this is the form most similar to normal feeding, and this is used if a patient has a functioning digestive tract and normal gastric emptying. It is the method of choice for conscious patients, especially for those who are walking and do not want to be subjected to a drip or infusion pump [19].

Based on the results of the panel, a tendency was found to use continuous EN for patients without constipation, and intermittent administration of EN was preferred in cases of constipation. This could be due to the stimulation of the gastrocolic reflex, which could be favoured by the intermittent administration of nutrition, thus improving constipation, although no available scientific evidence in this regard was found. 

There was no agreement among the panellists regarding the situation in which cyclic administration could be more beneficial than other forms of EN administration. In this regard, scientific evidence is also insufficient for making recommendations. 

### 4.3. Infusion Site

The indications and access routes for enteral nutrition for patients with hyperglycaemia are similar to those for patients without hyperglycaemia, and no specific recommendations have been found in this regard for this type of patient receiving EN.

Coinciding with the published literature [3,21], the panellists agreed that, in most clinical scenarios, the most appropriate EN infusion site for patients with hyperglycaemia is gastric infusion, except in the case of diabetic gastroparesis, where there was agreement on the use of post-pyloric infusion. 

### 4.4. Treatment of Diabetes

For outpatients with hyperglycaemia receiving EN, drug treatment can follow the same recommendations and clinical practice guidelines as any other patient with hyperglycaemia (for both insulin and other non-insulin antidiabetic agents) [3], although it is true that there is very little literature on the administration of oral antidiabetic drugs using a feeding tube, and the safety of crushing and administering antidiabetic drugs through a feeding tube is not always known. 

Regarding the use of metformin, the expert consensus results are consistent with current clinical practice guidelines. Metformin is a safe and effective drug that can reduce the risk of cardiovascular events [22]. Experts recommend assessing the clinical situation of a patient prior to using metformin. 

Regarding sulfonylureas, there was consensus in considering their use as inappropriate in cases with associated comorbidities, such as heart failure, liver failure, kidney failure, or cardiovascular risk, as well as for patients with BMIs of over 40 kg/m^2^. With sulfonylureas, there is associated weight gain, so their use is not recommended in cases of patients with high BMIs, even if there is associated malnutrition. As for long-acting sulfonylureas, they are not recommended in the guidelines for administration of medications by feeding tube [23]. 

There is no agreement in most clinical scenarios for the use of glinides. This lack of agreement is likely due to the lack of evidence of and experience with their use through enteral feeding tubes. In addition, they are considered inappropriate in cases of liver failure or in the case of a patient with a BMI of over 40 kg/m^2^ due to the weight gain associated with these drugs. Regarding the opinions of the experts in this study, there could be room for its use in patients receiving EN through bolus administration in the absence of other, more appropriate pharmacological options. There was no scenario that was agreed to be appropriate for the use of sulfonylureas or glinides.

In inpatients with hyperglycaemia, the best corrective treatment is insulin [7]. The insulin regimen to be used is the one that best suits the EN infusion method (continuous, cyclical or intermittent/bolus). The total dose of insulin required may be divided into basal insulin, prandial insulin and an insulin correction regimen.

In continuous EN, the guidelines recommend the use of basal insulin (glargine in a single daily dose, detemir in two daily doses, or NPH in two to three daily doses), together with prandial insulin, preferably every 8 h, with regular rapid-acting insulin and a regular rapid-acting insulin correction regimen [3,24]. The experts expressed agreement on the appropriate use of basal insulin glargine together with a bolus of regular rapid-acting insulin for patients with high glycaemia receiving continuous EN, and they also agreed on the use of basal insulin glargine together with a rescue regimen, with regular rapid-acting insulin in continuous EN for both controlled and high glycaemia. In the case of insulin detemir, the panel agreed on its use in combination with regular rapid-acting rescue insulin only in the case of continuous EN with high blood glycaemia without metabolic stress. There was no agreement on the rest of the situations involving the use of insulin detemir for patients with continuous EN, likely because, in these cases, two doses of insulin detemir would be required compared to a single dose of glargine. There was agreement on the inappropriate use of ultra-rapid-acting insulin in continuously administered EN, both in fixed and rescue regimens. In addition, the exclusive use of rapid-acting insulin (both regular rapid-acting and ultra-rapid-acting insulin) in a bolus without an associated basal component in continuous EN was considered inappropriate.

Insulin glargine as basal insulin for exclusive use without a fixed regimen of rapid-acting insulin or a corrective regimen does not conform to standard clinical practice, although the experts agreed on its use for patients receiving continuous EN with controlled glycemia and without associated metabolic stress, believing that it could be an appropriate guideline in hospitals for chronic patients or nursing homes. However, it is considered inappropriate in situations of high glycaemia or metabolic stress, and in these conditions, a corrective regimen of rapid-acting insulin, at minimum, should always be considered. 

For cyclically administered EN, the guidelines recommend the use of an intermediate-acting basal insulin, such as NPH or detemir, to be injected between half an hour and one hour before the infusion of EN, together with a corrective regimen of regular rapid-acting or ultra-rapid-acting insulin every 4–8 h [3]. The experts agreed on the appropriate use of insulin glargine as a basal component in cyclic EN, together with regular rapid-acting insulin as a fixed or rescue regimen for most clinical scenarios. In the case of insulin detemir, together with regular rapid-acting rescue insulin, its use in cyclic EN was agreed upon for cases of high glycaemia without metabolic stress, while no agreement was reached for the rest of the clinical scenarios for the use of insulin detemir in cyclic EN.

The regimen of administering insulin detemir as the exclusive basal component is considered inappropriate for most clinical situations, although this regimen could perhaps be useful for cyclic EN administered within 12 h as long as the patient has controlled glycemia without associated metabolic stress, although there was no agreement among the panellists. 

Regarding EN administered in bolus or intermittently, the guidelines recommend the use of a basal-bolus insulin regimen using ultra-rapid-acting insulin as the best prandial insulin in these patients [3]. The panellists agreed that the use of boluses of ultra-rapid-acting insulin as prandial or rescue insulin would only be indicated in intermittently administered EN. Despite what is recommended by the guidelines for the administration of EN in a bolus, the experts also agreed that the use of regular rapid-acting insulin as a prandial or rescue component is correct, and this was likely due to what was stated above about the scant experience with the use of ultra-rapid-acting insulin in EN. 

Insulin degludec is an ultra-long-acting basal insulin analogue, but there is little experience to date on its use for inpatients. There is only one study that has assessed the impact of insulin degludec on inpatients receiving nutritional support, and it concluded that insulin degludec has the potential to maintain stable glycaemic control and reduce glycaemic variability in these patients; however, it was an observational study with a small sample size [25]. The experts agreed only on its use together with regular rapid-acting insulin for rescue for patients with metabolic stress who receive continuous EN, regardless of glycaemic control, and together with bolus-administered regular rapid-acting insulin in cyclic EN with high glycaemia and metabolic stress. Further good quality studies are required to assess the safety and efficacy of this insulin for inpatients receiving medical nutrition therapy. 

The use of only regular rapid-acting insulin without associated basal insulin was difficult for the experts to agree on. In most clinical scenarios, this is a guideline that is considered inappropriate by experts, especially in the case of high blood glycaemia and metabolic stress. Its use could be considered for patients with controlled glycaemia receiving intermittent or cyclic EN, although with respect to these cases, there was no consensus among the panellists. The use of ultra-rapid-acting insulin in a bolus without an associated basal component was regarded as to be inappropriate for all clinical scenarios.

### 4.5. Management of Gastrointestinal Complications

The gastrointestinal complications associated with EN in patients with hyperglycaemia are similar to those in patients without this condition, and an appropriate choice of the type of formula, the route and form of administration, the infusion time and the volume of the doses can considerably reduce these complications [26].

Diarrhoea is one of the most frequent complications in patients receiving EN [27]. The panellists were in favour of using a formula with a higher percentage of soluble fibre for patients with hyperglycaemia and diarrhoea, opting for a specific normal-calorie and normal-protein formula for diabetes with a high percentage of soluble fibre for patients with HbA1c levels over 8% or high glycaemia, with no other associated comorbidities. In cases where HbA1c levels are under 8% or for controlled glycaemia, there was no consensus reached on the use of this type of formula, which could be explained because, for patients with adequate glycaemic control, the use of a non-specific formula for diabetes with a high soluble fibre content could also be considered. 

For the control of diarrhoea, high-calorie and high-protein diabetic formulas are more inappropriate, as they have a higher osmolarity. They are also inappropriate for patients with kidney failure due to protein intake and in cases of gastroparesis due to fibre, as previously mentioned in the first chapter. In no scenario was there agreement on their proper use in the context of diarrhoea. 

There was no consensus on the use of peptide formulas in cases of diarrhoea. The panellists reported that they would consider whether there is adequate glycaemic control, and they would also consider their use in situations such as for patients with pancreatitis, where the reduction in the percentage of fats and partial replacement with medium chain triglycerides (MCT) can improve diarrhoea symptoms.

The specific formulas for diabetes always contain fibre, which is why the panel considered them to be appropriate for most clinical scenarios associated with constipation. There was only agreement on the inappropriate use of a specific high-calorie and high-protein formula for diabetes in the context of constipation, together with kidney failure or gastroparesis, and this was due to the previously mentioned protein and fibre content. The experts agreed that the use of a peptide formula (without fibre) when constipation is present is inappropriate.

In the case of the coexistence of constipation and gastroparesis, the scenarios are uncertain since these are opposite situations in terms of whether or not to choose a high fibre content. For these patients, the opinions of the experts were to customise the decision in each case, although there is no scientific evidence to support this.

## 5. Limitations and Strengths

The steering and research group recognises the limitations and strengths of this panel of experts. The limitations include the difficulty encountered in its design, given the large number of variables used to build the proposed scenarios. The large sum of scenarios examined—a total of 2992—may have been confusing for the panellists, increasing the possibility of error in their interpretations. However, this number of clinical scenarios reflects the variability in the clinical reality that professionals face when making decisions about EN for patients with hyperglycaemia, and it makes it unlikely that we will encounter clinical situations that were not considered, which is a strength.

Another limitation to highlight is the representativeness of the group of experts, which was limited to a geographical region where there may have been influence from certain conditions outside clinical practice, such as the availability of formulas and cost. However, the selected panellists were professionals with more than 10 years of experience in clinical nutrition and, specifically, enteral nutrition, both for inpatients and outpatients. 

In the literature search, we did not find information to support the administration of dipeptidyl peptidase 4 (DPP-4) inhibitors or sodium-glucose co-transporter-2 (SGLT2) inhibitors by tube feeding; thus, we did not include these drugs in the clinical scenarios.

One of the greatest strengths is the methodology used. The RAND/UCLA Appropriateness Method is designed to detect scenarios in which there is consensus, but it does not force panellists to reach one. The criteria applied to analyse the results allowed the panellists to not agree or even to disagree on a series of scenarios or indications. Through the iterative process of multiple rounds, this method allowed the panellists to receive feedback from each other while maintaining anonymity at all times. With these fundamental aspects of the method, the opinions of the panellists converged much more easily towards opinions that were shared by many members of the panel.

## 6. Conclusions

The management of EN in patients with diabetes or stress hyperglycaemia is a major clinical challenge. Despite the high incidence of this pathology in patients who are candidates for EN, there are still many gaps in the current knowledge. The results of the panel showed that there are many areas of uncertainty, which opens up an enormous field for the future development of research projects, both for observational and randomised clinical trials.

The most important gaps were found in the choice of the most appropriate type of EN formula for each clinical scenario, as well as in determining the method of administration and the ideal treatment for each clinical situation.

However, the development of the panel of experts has contributed to establishing some recommendations on appropriate or inappropriate use in clinical situations where there is a lack of evidence, constituting a useful instrument for improving daily clinical practice in the management of EN in hyperglycaemia. 

There is still a lot of work to be completed in this field to ensure that clinical decisions are supported by an adequate level of evidence. Clinical trials on the efficacy of different types of formulas in various clinical situations are especially necessary, as are studies on optimising the treatment of hyperglycaemia in EN.

## Figures and Tables

**Table 1:** Summary of the results from chapter 1: types of formulas used.

Appropriate	Inappropriate
Use of a normal-calorie and normal-protein diabetes specific formula in stable outpatients Use of a high-protein and normal-calorie diabetes-specific formula in unstable inpatients with no other comorbidities The presence of PU determines the appropriate indication of a high-protein formula or a PU-specific formula The presence of constipation makes it appropriate to use a standard formula with fibre if there is good glycaemic control in stable patients (for both outpatients and inpatients) The use of specific formulas for kidney disease is prioritised in all clinical scenarios The use of protein modules is appropriate when there is a PU or in the case of malnourished obese patients	High-protein and high-calorie diabetes specific formulas: ○ By protein content *: ▪ In pre-dialysis kidney disease in almost all clinical outpatient and inpatient scenarios; exception: stressful situations, especially in malnourished patients ○ By calorie content *: ▪ Obese outpatients without malnutrition ▪ Stable, obese inpatients Standard formulas with/without fibre: ○ Patients with poor glycaemic control ○ Fibre formulas are inappropriate in the case of gastroparesis, and formulas without fibre are inappropriate in the case of constipation

PU: pressure ulcer. * Plausible explanation of the results obtained according to the opinions of the panel experts.

**Table 2:** Summary of the results from chapter 2: method of administration.

Appropriate	Inappropriate
Intermittent EN administration in almost all outpatients and stable inpatients Continuous administration is appropriate in the most unstable patients Panellists showed a trend to consider intermittent administration more appropriate for inpatients in the case of constipation There was no agreement on cyclical management in any scenario	Intermittent EN administration in the case of gastroparesis for all clinical scenarios

EN: enteral nutrition.

**Table 3:** Summary of the results from chapter 3: infusion site.

Appropriate	Inappropriate
The indications and routes of access for enteral nutrition in patients with hyperglycaemia are similar to those in patients without hyperglycaemia *	
Gastric infusion of enteral nutrition in all clinical scenarios except gastroparesis Post-pyloric infusion of enteral nutrition in patients with gastroparesis	Gastric infusion in gastroparesis

* Opinion of the panel members.

**Table 4:** Summary of the results from chapter 4: treatment of hyperglycaemia.

	Appropriate	Inappropriate
Oral Antidiabetic Drugs	Metformin: most clinical scenarios: ○ <70 years old, with no comorbidities or with CV risk ○ >70 years old, with no comorbidities and with a BMI of >40 or with CV risk Sulfonylureas: none Glynides: none scenario. According to the experts, there could be a place for their use in EN administered in bolus (no consensus) *	Metformin: usual contraindications: >70 years old with kidney or liver disease Sulfonylureas: the usual contraindications: heart failure, liver or kidney disease, CV risk, or a BMI of >40 Glinides: liver disease and a BMI of >40
Insulin	In continuous/cyclic EN: ○ Insulin glargine in almost all clinical situations evaluated adding: ▪ Regular rapid-acting insulin as a fixed bolus if high glycaemia ▪ Extra bolus of rapid-acting regular insulin according to glycaemic level ○ Insulin glargine without associated rapid-acting insulin regimen in stable patients (continuous EN) ○ Insulin detemir combined with an extra bolus of rapid-acting regular insulin according to glycaemic levels ○ Insulin degludec adding: ▪ Extra bolus of rapid-acting regular insulin according to glycaemic level Rapid ▪ Regular rapid-acting insulin as a fixed bolus in cyclic EN if high glycaemia and metabolic stress In intermittent EN: ○ Insulin glargine in almost all clinical situations, adding: ▪ Regular rapid-acting insulin as a fixed bolus if high glycaemia ▪ Extra bolus of rapid-acting regular insulin according to glycaemic level ○ Insulin glargine in almost all clinical situations, adding: ▪ Ultra-rapid-acting insulin as a fixed bolus if high glycaemia and metabolic stress (unstable patient) ▪ Extra bolus of ultra-rapid-acting insulin if controlled glycaemia with or without stress or high glycaemia without stress ○ Insulin detemir; adding rescue insulin: ▪ Extra bolus of rapid-acting regular insulin if controlled glycaemia and metabolic stress or high glycaemia without metabolic stress ▪ Extra bolus of ultra-rapid-acting insulin if high glycaemia and metabolic stress	In continuous EN: ○ Regular/ultra-rapid-acting insulin in a fixed bolus exclusively In intermittent EN: ○ Insulin glargine exclusively without fixed or corrective regimen in the case of controlled glycaemia with stress fixed ○ Insulin detemir exclusively in a single dose for high glycaemia or metabolic stress With any method of administration of EN: ○ Any exclusively basal insulin without a fixed or corrective regimen in the case of high glycaemia and metabolic stress ○ Regular insulin as a fixed bolus without basal insulin in the case of high glycaemia and metabolic stress ○ Ultra-rapid-acting insulin without basal insulin in any scenario

CV: cardiovascular; BMI: body mass index; EN: enteral nutrition; * Plausible explanation of the results obtained according to the opinions of the panel experts.

**Table 5:** Summary of the results from chapter 5: management of complications of enteral nutrition.

	Appropriate	Inappropriate
Diarrhoea	Specific normal-calorie and normal-protein formula for diabetes (with a high percentage of soluble fibre) for patients with poor glycaemic control and no associated comorbidity (for both outpatients (HbA1c > 8%) and inpatients with normal nutrition (high glycaemia))	Specific high-calorie and high-protein formulas for diabetes (higher osmolarity *) for patients with kidney failure (due to protein content *) and in the case of gastroparesis (due to fibre *) (for both outpatients and inpatients) Standard formula (with or without fibre) for patients with poor glycaemic control (HbA1c >8% or high glycaemia); the formula with fibre would be inappropriate in the case of gastroparesis, regardless of glycaemic control Standard formula with fibre for patients with good glycaemic control but with pressure ulcers or kidney failure
Constipation	Specific normal-calorie and normal-protein formula for diabetes for patients with normal nutrition and no comorbidity (both for outpatients (regardless of glycaemic control) and inpatients (with high glycaemia and without stress)) Specific high-calorie and high-protein formula for diabetes for: ○ Outpatients with PU and inpatients with PU (normonourished and without stress) ○ With high glycaemia and stress without PUs Standard formula with fibre for outpatients with normal nutrition, who are over 70 years old, without comorbidity, and with HbA1c < 8% (stable elderly patients)	Specific high-calorie and high-protein diabetes formula for patients with kidney failure (for protein content *), for patients with normal nutrition (due to calorie content *), and in cases of gastroparesis (due to fibre content *) Standard formula (with or without fibre) for patients with HbA1c >8% or high glycaemia; the formula with fibre would be inappropriate in the case of gastroparesis Use of standard formula with fibre for patients with controlled glycaemia but with gastroparesis (due to fibre content *) or PU (due to protein content *) Peptide formula (without fibre)

HbA1c: glycosylated haemoglobin. PU: pressure ulcer. * Plausible explanation for the results obtained according to the opinions of the panel experts.

## Data Availability

The full information on the scores for all scenarios is available upon request to the corresponding author.

## Appendix A

- Calorie-protein and vitamin-mineral targets:
  - Do your patients with diabetes mellitus (DM) receive different calorie and/or protein intake through enteral nutrition (EN) than those without DM? If the answer is yes, state why;
  - What type of formula (or formulas) would you use to prescribe a low-calorie EN?/high-protein for patients with DM and body mass index (BMI) > 40 kg/m^2^?
- Administration regimen:
  - Regarding the starting administration regimen (not the type of formula). Does the fact that the patient has DM change anything? Respond yes or no and justify the answer;
  - Regarding subsequent regimens, does the fact that the patient has DM change anything? Respond yes or no and justify the answer;
  - Which method of administration would you prefer to use in patients with DM? Continuous 24 h, continuous day/night or gravity/bolus. Justify the answer.
- Specific formulas for diabetes or stress hyperglycaemia:
  - Do you always use “specific formulas for diabetes” as the first option in people with DM or stress hyperglycaemia? Respond yes or no and justify the answer;
  - Would you use specific formulas for DM in prediabetes? Respond yes or no and justify the answer;
  - In a patient with DM and home enteral nutrition (HEN), do you consider HbA1c to be useful data for deciding to prescribe standard or specific formulas? Respond yes or no and justify the answer;
  - In an inpatient with stress hyperglycaemia or DM with overt hyperglycaemia (mean glycaemia around 180 mg/dl), would you prescribe a specific enteral formula for DM? Respond yes or no and justify the answer;
  - Do you think that standard formulas with fibre can be used in home nutrition in people with DM? Respond yes or no and justify the answer;
  - In inpatients with DM who are discharged home with HEN, do you maintain the same formula? Why?
  - Would you use other specific formulas for patients with DM (oligopeptide/low-fat, immunomodulatory, with Omega-3, beta-hydroxy-beta-methylbutyrate (HMB), etc.)? Specify in which clinical situations you would prescribe them;
  - Regarding the composition of the formulas in people with DM:
    - Regarding the concept of “formula for people with diabetes or stress hyperglycaemia”, what is the aim with its chemical formulation?
    - What do you think is the ideal distribution of macronutrients?
    - Would you use fructose?
    - What proteins would you use?
    - Would you supplement with “extra” doses of micronutrients (above the Dietary Reference Intakes (DRIs)). If yes, which?
    - Is a fibre-free formula beneficial in people with DM? Justify each answer;
  - The price of formulas for people with DM is higher than the standard equivalents:
    - Do you know by approximately how much?
    - Do you think it is justified to use formulas for diabetes, despite the increased cost for public health? Would it be cost-effective?
    - Do you take price into account when prescribing? Respond yes or no and justify the answer.
- Specific formulas in various clinical situations:
  - Person with DM with HEN with a specific formula for diabetes and constipation. What would you do? Justify the answer;
  - Neurological patient, with DM, bedridden and malnourished with HEN with a specific formula that presents abdominal distension and meteorism, do you think it is appropriate to maintain the same formula? Justify the answer;
  - Bedridden DM patient with HEN and high risk of pressure ulcers. Should they be treated preventively with arginine supplements or with a higher protein content than current formulas for DM? Justify the answer;
  - For patients with DM and gastrointestinal cancer, what type of enteral formula would you use in the perioperative period? Justify the answer;
  - For patients with DM and head and neck cancer, what type of enteral formula would you use in the perioperative period? Justify the answer;
  - For patients with DM and predialysis or dialysis chronic kidney disease (CKD) requiring EN by tube, what kind of formula would you use? Justify the answer;
  - For patients with DM who presents diarrhoea attributable to EN itself, what type of formula would you use? Justify the answer;
  - For patients with DM and hypertriglyceridemia over 400 mg/dl, what kind of formula would you use? Justify the answer;
  - For patients with DM and EN by nasogastric tube presenting gastroparesis, what formula would you use? Justify the answer;
  - In case of severe gastroparesis that requires infusion of nutrition into the jejunum; what formula would you use? Justify the answer;
  - In what situations do you consider it necessary to use micronutrient supplementation in people with DM? Justify the answer.
- Treatment of diabetes in EN:
  - What would be the ideal oral hypoglycaemic agents (OHAs) treatment in patients with DM2 and HEN?
  - Do the new drugs (GLP-1-1 analogues; SGLT-2) have a role in the management of patients with EN and DM2?
  - Do you use the glycaemic load calculation and the sensitivity index to calculate bolus insulin in people with a bolus or who are critically ill? Respond yes or no and justify the answer;
  - Which insulin do you consider most appropriate as basal insulin? As night-time or daytime EN, as continuous 24-h EN and as EN in bolus or by gravity. Justify the answer;
  - Which insulin do you consider most appropriate as bolus insulin? As night-time or daytime EN, as continuous 24-h EN and as EN in bolus or by gravity. Justify the answer;
  - Which insulin do you consider most appropriate as a correction or rescue insulin? As night-time or daytime EN, as continuous 24-h EN and as EN in bolus or by gravity. Justify the answer;
  - What do you think influences you the most when prescribing EN to a patient (select the top three):
    - Type of formula: macronutrient composition;
    - Formula type: calorie density;
    - Formula Type: flavouring;
    - Price;
    - Characteristics of the container (size, weight, grip, opening and closing, etc.) and connections;
    - Patient preferences;
    - Marketing of the manufacturing company;
    - Clinical studies of the formula.

## Appendix B

- Variables used to create the scenarios in chapters 1, 2 and 3:
  - Patient point of care: outpatient or inpatient;
  - Age (only in outpatients): under 70 years or 70 years or older;
  - Glycaemia (only in inpatients): controlled or high;
  - Metabolic stress (only in inpatients): without stress or with stress;
  - Glycaemia (only in outpatients): glycosylated haemoglobin greater than 8% or glycosylated haemoglobin under 8%;
  - Nutritional status: malnourished or normonourished;
  - Body Mass Index (BMI): BMI < 40 kg/m^2^ or BMI > 40 kg/m^2^;
  - Kidney failure (KF) (only in patients with BMI < 40): without kidney failure (KF) or with KF;
  - Comorbidity (only in patients with BMI < 40 and without KF): no comorbidities, gastroparesis, pressure ulcer or constipation.
- Variables used to build the scenarios in chapter 4 (for antidiabetic drugs):
  - Patient point of care: outpatient;
  - Age: under 70 years or 70 years or older;
  - Glycaemia: glycosylated haemoglobin greater than 8% or glycosylated haemoglobin under 8%;
  - Nutritional status: malnourished or normonourished;
  - Body Mass Index (BMI): BMI < 40 kg/m^2^ or BMI > 40 kg/m^2^;
  - Comorbidity: No comorbidities, kidney failure, liver failure, heart failure or cardiovascular risk.
- Variables used to create the scenarios in chapter 4 (for insulins):
  - Patient point of care: inpatients;
  - Glycaemia: controlled or high;
  - Metabolic stress: without stress or with stress;
  - Nutritional status: malnourished or normonourished;
  - Body Mass Index (BMI): BMI < 40 kg/m^2^ or BMI > 40 kg/m^2^;
  - Enteral nutrition form of administration: continuous or bolus/intermittent or cyclic administration.
- Variables used to build the scenarios in chapter 5:
  - Patient point of care: outpatient or inpatient;
  - Age (only in outpatients): under 70 years or 70 years or older;
  - Glycaemia (only in inpatients): controlled or high;
  - Metabolic stress (only in inpatients): without stress or with stress;
  - Glycaemia (only in outpatients): glycosylated haemoglobin greater than 8% or glycosylated haemoglobin under 8%;
  - Nutritional status: malnourished or normonourished;
  - Side effects: no comorbidities, gastroparesis, pressure ulcer or kidney failure.

## Appendix C

**Term**	**Definition**
Cyclic administration	Administration at a constant rate of enteral nutrition (EN) with a pump for a period of 8–12 h. (it can be during the day with a break at night or vice versa)
Continuous administration	Administration at a constant rate of EN with a pump, without interruption over 24 h. (also acceptable if there is a 1–2 h break)
Bolus/intermittent administration	Bolus administration of EN in 3–6 doses per day. It can be with a syringe or by gravity, with a duration of 30 to 120 min per dose.
Degludec	Insulin analogue over more than 24 h.
Malnutrition	Clinical situation caused by a nutrient deficit (due to inadequate intake, increased losses, increased requirements, altered absorption and/or inflammation) that entails a change in body composition and that decreases physical and mental functions and has a negative impact on the clinical evolution of the patient.
Disease-related malnutrition	Malnutrition caused directly or indirectly by suffering from an acute, subacute or chronic disease.
Detemir	Long-acting insulin analogue (16–24 h)
Diarrhoea	Producing loose or liquid stools, three or more times a day (or with a greater than normal frequency for the person).
Distension	Feeling of fullness, nausea, bloating or abdominal pain, due to the accumulation of gas in the stomach and intestine, or accumulation of abdominal fluid.
Constipation/equivalent to chronic constipation	less than 3 bowel movements a week and/or hard stools in more than 25% of bowel movements, or a feeling of incomplete or very difficult defecations.
Low-fat formula	Enteral formula with fat content 5–20% of the total energy (TE).
High-calorie/high-protein diabetic formula	Complete polymeric enteral formula with density >1.2 Kcal/mL, protein content >18% of TE, which contains fermentable fibre in a high or exclusive proportion, low glycaemic index carbohydrates, and/or prebiotics (fructooligosaccharides (FOS), Inulin) and, frequently, a high content of monounsaturated fatty acids.
Normal-calorie/high-protein diabetic formula	Complete polymeric enteral formula with density >0.9 and <1.2 Kcal/mL, protein content ≥18% of TE, which contains fermentable fibre in a high or exclusive proportion, carbohydrates, which contain soluble fibre in a high or exclusive proportion. Low glycaemic index carbohydrates, and/or prebiotics (FOS, Inulin) and, frequently, a high content of monounsaturated fatty acids.
Normal-calorie/normal-protein diabetic formula	Complete polymeric enteral formula with density >0.9 and <1.2 Kcal/mL, protein content 11–18% of TE, which contains fermentable fibre in a high or exclusive proportion, low glycaemic index carbohydrates, and/or prebiotics (FOS, Inulin) and, frequently, a high content of monounsaturated fatty acids.
Standard formula	Complete polymeric enteral formula with density >0.9 and <1.2 Kcal/mL, protein content 11–18% of TE, which do not contain fibre.
Standard formula with fibre	Complete polymeric enteral formula with density >0.9 and <1.2 Kcal/mL, protein content 11–18% of TE, which contain fermentable, non-fermentable fibre or a mixture of fibres.
Immunomodulatory formula	Complete enteral formula, protein content >18% of TE, enriched to varying degrees with: glutamine, Omega-3 fatty acids, arginine, nucleotides, micronutrients.
Peptide formula	Complete oligomeric enteral formula, which contains hydrolysed proteins (peptides), in addition to carbohydrates, fats with high medium chain triglycerides (MCT) and micronutrient content.
VLCD(very low calorie diet) formulas	Omega-3 fatty acids, arginine, nucleotides, micronutrients.
Specific formulas	Complete enteral formula with a nutrient profile appropriate to metabolic situations or requirements of a specific pathology (diabetes and stress hyperglycaemia, nephropathy, liver disease, stress or immunosuppression, oncology, respiratory failure, cystic fibrosis).
Gastroparesis	Delayed gastric emptying observed in the absence of gastric obstruction, and accompanied by early satiety, nausea or vomiting, or abdominal pain.
Glargine U100 and U300	Long-acting insulin analogue (U100 20–24 h U300 over 24 h).
Glinides or meglitinides	Group of oral hypoglycaemic drugs that increase insulin secretion.
HbA1c	Glycosylated haemoglobin.
Hyperglycaemia	Glycaemia ≥ 126 mg/dL.
Body Mass Index (BMI)	Degree of adiposity calculated according to the formula body weight (Kg)/height (m^2^).
Gastric infusion	EN infusion through a tube whose distal end is placed in the stomach.
Postpyloric infusion	Infusion of EN through a tube whose distal end is placed in the duodenum or jejunum.
Bolus and rescue insulin	Insulin administration in preset doses with established intervals (every 6–8 h, or prandial), adding a higher dose to the preset dose if glycaemia exceeds a preset limit.
Rapid-acting insulin	Regular insulin duration (5–7 h).
Ultra-rapid-acting insulin	Insulin analogues (Lispro, aspart, glulisine) duration (2–4 h).
Malabsorption	Signs and symptoms derived from a deficit in the intestinal absorption of nutrients.
Metformin	Oral hypoglycaemic drug from the biguanide group that reduces postprandial and basal glycaemia.
Normal nutrition	Normal nutritional status according to assessment scales such as subjective global assessment (SGA) or clinical and analytical assessment methods.
NPH	Intermediate-acting insulin (12–16 h).
Obesity	BMI ≥ 30 k/m^2^.
Outpatient	Patient who receives health care while living in the community (care home or private residence) and who comes to the medical or nursing appointment in person or virtually.
Inpatients	Patient who remains admitted to hospital for diagnosis or treatment.
Metabolic stress	Metabolic response to trauma, sepsis, surgery that is associated with increased energy expenditure and muscle catabolism manifested by hyperglycaemia increased urinary nitrogen losses and oxygen consumption.
Sulfonylurea	Group of oral hypoglycaemic drugs that increase insulin secretion and boost the action of insulin in extrapancreatic tissues.
Pressure ulcer	Localised area of damage to the skin and underlying tissues caused by pressure, friction, or a combination of both.

## Appendix D

**Table A1:** Main agreements detected in the panel from chapter 1: types of formulas used.

Appropriate Indication
Use of a specific formula for diabetes in patients with hyperglycaemia: Outpatients: High-protein and normal-calorie in PU; High-protein and high-calorie in PU, except in normonourished patients; Normal-protein and normal-calorie in outpatients, normonourished and without associated comorbidity (regardless of glycaemic control). Inpatients: High-protein and normal-calorie in PU; High-protein and high-calorie in PU, except in normonourished patients; High-protein and normal-calorie, in the context of high glycaemia and metabolic stress, normonourished with a BMI under 40 kg/m^2^ and without associated comorbidity.
2. Use of a standard formula with fibre in a patient with hyperglycaemia and constipation: Outpatients, under 70 years old, normonourished, with HbA1c under 8%. Inpatients, normonourished, with controlled glycaemia and without metabolic stress.
3. Use of a specific formula for kidney failure in patients with hyperglycaemia and kidney failure.
4. Use of a specific formula for PU in patients with hyperglycaemia and PU.
5. Use of protein modules in patients with hyperglycaemia: Outpatients: Malnourished with PU, regardless of metabolic control and age; Malnourished with a BMI over 40 kg/m^2^, regardless of metabolic control. Inpatients: With PU, regardless of nutritional status and metabolic control.
**Inappropriate Indication**
Use of a specific high-calorie and high-protein formula for diabetes in patients with hyperglycaemia: Outpatients: With pre-dialysis kidney failure; Under 70 years old, normonourished, with a BMI over 40 kg/m^2^ (regardless of glycaemic control); With HbA1c under 8% and gastroparesis, regardless of nutritional status and age. Inpatients: With pre-dialysis kidney failure, except in patients with metabolic stress; With controlled glycaemia and without metabolic stress and a BMI over 40 kg/m^2^ (regardless of nutritional status); With gastroparesis, regardless of nutritional status.
2. Use of a specific high-protein and normal-calorie formula for diabetes in patients with hyperglycaemia: Inpatients: With pre-dialysis kidney failure, and with controlled glycaemia, without metabolic stress (regardless of nutritional status).
3. Use of a standard formula without fibre in patients with hyperglycaemia: Outpatients: With constipation; With HbA1c over 8%. Inpatients: With constipation; With high glycaemia.
4. Use of a standard formula with fibre in a patient with hyperglycaemia: Outpatients: With gastroparesis; With HbA1c over 8%. Inpatients: With gastroparesis; With high glycaemia.
5. Use of protein modules in patients with hyperglycaemia: Outpatients: Normonourished, under 70 years old, with a BMI under 40 kg/m2 and without associated comorbidity (regardless of glycaemic control). Inpatients: Normonourished, with controlled glycaemia and without metabolic stress, without associated comorbidity and with a BMI under 40 kg/m^2^.

PU: pressure ulcer. BMI: body mass index. HbA1c: glycosylated haemoglobin. In the first chapter, 18% were appropriate scenarios, 10% inappropriate and 72% uncertain.

**Table A2:** Main agreements detected in the panel from chapter 2: method of administration.

Appropriate Indication
Use of EN in continuous administration in patients with hyperglycaemia: Outpatients: With gastroparesis. Inpatients: With gastroparesis; With high glycaemia and metabolic stress, without constipation (regardless of nutritional status).
2. Use of EN in intermittent administration in patients with hyperglycaemia: Outpatients: Without gastroparesis. Inpatients: Without metabolic stress and with constipation (regardless of glycaemic control or nutritional status).
**Inappropriate Indication**
Use of EN in continuous administration in patients with hyperglycaemia: Outpatients: Normonourished, except for gastroparesis; Malnourished, with HbA1c under 8% and with constipation.
2. Use of EN in intermittent administration in patients with hyperglycaemia: Outpatients and inpatients: With gastroparesis.

EN: enteral nutrition. HbA1c: glycosylated haemoglobin. In the second chapter, 10.4% were appropriate scenarios, 21.5% inappropriate and 68.1% uncertain.

**Table A3:** Main agreements detected in the panel from chapter 3: infusion site.

Appropriate Indication
Gastric infusion of EN in patients with hyperglycaemia: Outpatients and inpatients: Without gastroparesis.
2. Post-pyloric infusion of EN in patients with hyperglycaemia: Outpatients and inpatients: With gastroparesis.
**Inappropriate Indication**
Gastric infusion of EN in patients with hyperglycaemia: Outpatients and inpatients: With gastroparesis.
2. Post-pyloric infusion of EN in patients with hyperglycaemia: Outpatients: Without gastroparesis. Inpatients: With controlled glycemia, without metabolic stress and without associated comorbidities (regardless of nutritional status).

EN: enteral nutrition. In the third chapter, there were 19.8% scenarios considered appropriate, 42.2% inappropriate and 38.2% uncertain.

**Table A4:** Main agreements detected in the panel from chapter 4: treatment of hyperglycaemia.

Appropriate Indication
1. Use of metformin in patients with hyperglycaemia: Under 70 years old, without comorbidity or with cardiovascular risk (regardless of nutritional status, glycaemic control or BMI); Over 70 years old, BMI over 40 kg/m^2^, without associated comorbidity or with cardiovascular risk.
2. Insulin use in continuous EN in patients with hyperglycaemia: Basal insulin glargine together with boluses of regular rapid-acting insulin in case of high glycaemia; Basal insulin glargine together with a rescue regimen of regular rapid-acting insulin in the case of controlled or high glycaemia with metabolic stress and in the case of high glycaemia without metabolic stress; Exclusive glargine basal insulin without a fixed or corrective regimen of rapid-acting insulin in the case of controlled glycaemia and without metabolic stress; Insulin detemir together with a rescue regimen of regular rapid-acting insulin in the case of high glycaemia and without metabolic stress; Insulin degludec together with regular rapid-acting rescue insulin in case of metabolic stress, regardless of glycaemic control.
3. Insulin use in cyclic EN in patients with hyperglycaemia: Basal insulin glargine together with boluses of regular rapid-acting insulin in case of high glycaemia with metabolic stress; Basal insulin glargine together with a rescue regimen of regular rapid-acting insulin in the case of controlled or high glycaemia with metabolic stress and in the case of high glycaemia without metabolic stress; Detemir basal insulin together with a rescue regimen of regular rapid-acting insulin in the case of high glycaemia and without metabolic stress; Insulin degludec together with boluses of regular rapid-acting insulin in cases of high glycaemia and metabolic stress.
4. Insulin use in intermittent EN in hyperglycaemic patients: Basal insulin glargine together with boluses of regular rapid-acting insulin in case of high glycaemia; Basal insulin glargine together with boluses of ultra-rapid-acting insulin in the case of high glycaemia with metabolic stress; Basal insulin glargine together with a rescue regimen of regular rapid-acting insulin in the case of controlled glycaemia with metabolic stress and in the case of high glycaemia without metabolic stress; Basal insulin glargine together with ultra-rapid-acting rescue insulin in the case of controlled glycaemia regardless of metabolic stress and in the case of high glycaemia without metabolic stress; Insulin detemir together with regular rapid-acting rescue insulin in case of controlled glycaemia and with metabolic stress and high glycaemia without metabolic stress; Insulin detemir together with ultra-rapid-acting rescue insulin in the case of high glycaemia and metabolic stress.
**Inappropriate Indication**
1. Use of metformin in patients with hyperglycaemia: With liver failure; Over 70 years old and kidney failure.
2. Use of sulfonylureas in patients with hyperglycaemia: With associated comorbidity such as heart failure, liver failure, kidney failure or cardiovascular risk; No comorbidity associated with BMI over 40 kg/m^2^, regardless of nutritional status or glycaemic control.
3. Use of glinides in patients with hyperglycaemia: With liver failure or BMI over 40 kg/m^2^.
4. Insulin use in continuous EN in patients with hyperglycaemia: Insulin detemir together with boluses of ultra-rapid-acting insulin in case of controlled glycaemia and without metabolic stress; Rapid-acting insulin in bolus exclusively (both regular ultra-rapid-acting and rapid-acting insulin).
5. Insulin use in intermittent EN in hyperglycaemic patients: Insulin glargine as basal insulin exclusively without a fixed or corrective regimen of rapid-acting insulin in the case of controlled glycaemia and with metabolic stress; Insulin detemir exclusively in a single dose for controlled glycaemia with metabolic stress or high glycaemia regardless of metabolic stress.
6. Use of insulin in any method of EN administration in hyperglycaemic patients: Basal insulin glargine/detemir/degludec as an exclusive component without a fixed or corrective regimen of rapid-acting insulin in the case of high glycaemia with metabolic stress; Regular rapid-acting bolus insulin without associated basal component in case of high glycaemia and metabolic stress; Rapid-acting insulin without associated basal insulin.

EN: enteral nutrition. BMI: body mass index. In the fourth chapter, 25.6% of scenarios were appropriate, 12% inappropriate and 62.4% uncertain.

**Table A5:** Main agreements detected in the panel from chapter 5: management of complications.

Appropriate Indication
Use of a specific normal-calorie and normal-protein formula for diabetes in patients with hyperglycaemia: Outpatients: With diarrhoea, without associated comorbidity and with HbA1c over 8%; With constipation, without associated comorbidity and normonourished (regardless of glycaemic control). Inpatients: With diarrhoea, without associated comorbidity, normonourished and high glycaemia; With constipation, without associated comorbidity, normonourished with high glycaemia and without metabolic stress.
2. Use of a specific high-calorie and high-protein formula for diabetes in patients with hyperglycaemia: Outpatients: With constipation and PU, regardless of nutritional status or glycaemic control. Inpatients: With constipation and PU, without metabolic stress, normonourished; With constipation, high glycaemia with metabolic stress, without comorbidity or PU.
3. Use of a standard formula with fibre in diabetic patients or with stress hyperglycaemia: Outpatients: With constipation, normonourished, over 70 years old and with HbA1c under 8%, without comorbidity.
**Inappropriate Indication**
Use of a specific high-calorie and high-protein formula for diabetes in patients with hyperglycaemia: Outpatients: With diarrhoea, along with gastroparesis or kidney failure; With constipation and kidney failure; With constipation, normonourished and gastroparesis. Inpatients: With diarrhoea, along with gastroparesis or kidney failure; With constipation and kidney failure.
2. Use of a standard formula (without fibre) in patients with hyperglycaemia: Outpatients: With diarrhoea or constipation, HbA1c over 8%, without gastroparesis; With constipation, malnourished with HbA1c under 8%, along with kidney failure or PU. Inpatients: With diarrhoea or constipation and high glycaemia, without gastroparesis; With constipation, controlled glycaemia, normonourished without comorbidity or with PU.
3. Use of a standard formula (with fibre) in patients with hyperglycaemia: Outpatients: With diarrhoea and gastroparesis; With diarrhoea or constipation and HbA1c over 8%; With diarrhoea, HbA1c under 8% and PU or kidney failure. Inpatients: With diarrhoea and gastroparesis; With diarrhoea or constipation and high glycaemia; With diarrhoea, controlled glycaemia and PU; With constipation, controlled glycaemia, with gastroparesis or PU.
4. Use of a peptide formula (without fibre) in patients with stress hyperglycaemia: Outpatients and inpatients: With constipation.

PU: pressure ulcer. HbA1c: glycosylated haemoglobin. In the fifth chapter, 4.5% of scenarios were appropriate, 61.3% inappropriate and 34.2% uncertain.

## Appendix E

**Table A6:** Median, agreement and appropriateness in all scenarios after the second round.

Item	Result
Median (average)	4.3
Median absolute deviation	1.0
% Scenarios with agreement (*n*)	90.4 (2706)
% Scenarios with disagreement (*n*)	0.5 (14)
% Appropriate scenarios (*n*)	23.7 (708)
% Inappropriate scenarios (*n*)	12.7 (381)
% Uncertain scenarios (*n*)	63.6 (1903)

**Table A7:** Median, agreement and appropriateness in the chapter 1 scenarios, after the second round.

Item	Result
Median (average)	4.4
Median absolute deviation	1.0
% Scenarios with agreement (*n*)	83.7 (643)
% Scenarios with disagreement (*n*)	1.0 (8)
% Appropriate scenarios (*n*)	18.0 (138)
% Inappropriate scenarios (*n*)	10.0 (77)
% Uncertain scenarios (*n*)	72.0 (553)

**Table A8:** Median, agreement and appropriateness in the chapter 2 scenarios, after the second round.

Item	Result
Median (average)	5.1
Median absolute deviation	0.5
% Scenarios with agreement (*n*)	69.1 (199)
% Scenarios with disagreement (*n*)	0.0 (0)
% Appropriate scenarios (*n*)	10.4 (30)
% Inappropriate scenarios (*n*)	21.5 (62)
% Uncertain scenarios (*n*)	68.1 (196)

**Table A9:** Median, agreement and appropriateness in the chapter 3 scenarios, after the second round.

Item	Result
Median (average)	5.4
Median absolute deviation	3.0
% Scenarios with agreement (*n*)	84.9 (163)
% Scenarios with disagreement (*n*)	3.1 (6)
% Appropriate scenarios (*n*)	19.8 (38)
% Inappropriate scenarios (*n*)	42.2 (73)
% Uncertain scenarios (*n*)	38.2 (73)

**Table A10:** Median, agreement and appropriateness in the chapter 4 scenarios, after the second round.

Item	Result
Median (average)	4.3
Median absolute deviation	1.5
% Scenarios with agreement (*n*)	98.1 (1083)
% Scenarios with disagreement (*n*)	1.9 (21)
% Appropriate scenarios (*n*)	25.6 (283)
% Inappropriate scenarios (*n*)	12.0 (132)
% Uncertain scenarios (*n*)	62.4 (689)

**Table A11:** Median, agreement and appropriateness in the chapter 5 scenarios, after the second round.

Item	Result
Median (average)	3.7
Median absolute deviation	1.5
% Scenarios with agreement (*n*)	96.6 (618)
% Scenarios with disagreement (*n*)	3.4 (22)
% Appropriate scenarios (*n*)	4.5 (29)
% Inappropriate scenarios (*n*)	61.3 (392)
% Uncertain scenarios (*n*)	34.2 (219)
